# ﻿Additional new species of the genus *Zaitzevia* (Coleoptera, Elmidae) from China with an updated key to species from mainland China

**DOI:** 10.3897/zookeys.1258.169390

**Published:** 2025-11-05

**Authors:** Ri-Xin Jiang, Lin Yang, Jian-Kun Long, Zhi-Min Chang, Chuan-Liang Sun, Xiao Feng, Mao-Heng Du, Xiang-Sheng Chen

**Affiliations:** 1 Guizhou Key Laboratory of Agricultural Biosecurity, Guiyang 550025, Guizhou, China Guizhou Key Laboratory of Agricultural Biosecurity Guiyang China; 2 Institute of Entomology, Guizhou University, Guiyang, 550025, Guizhou, China Guizhou University Guiyang China; 3 The Provincial Special Key Laboratory for Development and Utilization of Insect Resources, Guiyang 550025, Guizhou, China The Provincial Special Key Laboratory for Development and Utilization of Insect Resources Guiyang China; 4 The Administrations of Fodingshan National Nature Reserve, Tongren, 554300, Guizhou, China The Administrations of Fodingshan National Nature Reserve Tongren China

**Keywords:** Aquatic beetle, checklist, China, Elmidae, identification key, Macronychini, new species, riffle beetle, taxonomy

## Abstract

The riffle beetle genus *Zaitzevia* Champion, 1923 comprises 29 described species, distributed across Asia and North America. China exhibits particularly high species diversity within this genus, with 16 recorded species. In this study, we describe two new *Zaitzevia* species from China: *Z. fodingshanus***sp. nov.** from Guizhou Province and *Z. lipingae***sp. nov.** from Yunnan Province. Habitus and diagnostic features of the new species are illustrated, along with a checklist of all known Chinese *Zaitzevia* species, a key, and a distribution map of *Zaitzevia* species from mainland China. The comparative diagnoses discuss characters of the new and known species. The results show that the existing species diversity requires more detailed research acrosslarger areas of Southwest China in the future.

## ﻿Introduction

The Macronychini genus *Zaitzevia* Champion, 1923 comprises 29 valid species, distributed across East, Southeast, and Central Asia as well as North America (Jäch et al. 2016; Jiang and Wang 2021; Bian and Zhang 2022). Members of this genus are characterized by the extremely short antennae, which are divided into eight segments with the apical segment strongly expanded, and the elytra bearing granulate carinae on strial intervals 5, 7, and 8 (Brown 2001).

The Japanese fauna of *Zaitzevia* was recently reviewed (Iwata et al. 2022), and recent studies have revealed particularly high species diversity of this genus in China, with 16 species recorded prior to the present study (Jiang and Wang 2020, 2021; Bian and Zhang 2022; Jiang and Chen 2023; Bian and Hu 2024; Peng et al. 2024). However, the true diversity of the genus remains incompletely understood, with numerous species still awaiting description (Jäch and Boukal 1995; Jiang and Chen 2023).

As part of our ongoing aquatic beetle survey in Guizhou Province initiated in 2021, a series of new elmid beetles have been discovered and described (Jiang et al. 2023, 2024; Jiang and Chen 2025a, 2025b). Recently, we have commenced a new phase of investigation in collaboration with the administrations of nature reserves across Guizhou Province, and have made several new discoveries with their support. In this study, we describe two new *Zaitzevia* species: *Z. fodingshanus* sp. nov. from Guizhou Province, China and *Z. lipingae* sp. nov. from Yunnan Province, China. Diagnoses, descriptions, and illustrations of the new species are provided, along with a complete checklist of all known *Zaitzevia* species recorded from China and a key and a distribution map (Fig. 7) to known *Zaitzevia* species from mainland China.

## ﻿Material and methods

The material examined during this work is deposited in the Institute of Entomology, Guizhou University, Guiyang, China (**GUGC**).

Collecting data on the specimens are quoted verbatim. The Chinese translation of each locality below the provincial level is included in parentheses at the first appearance in the text. Each type specimen bears the following label: ‘HOLOTYPE (red) (or PARATYPE (yellow)), ♂, *Zaitzevia* + specific name sp. nov., Jiang & Chen, 2025.

Habitus images were taken using a Canon 5D Mark IV digital camera with an MP-E 65 mm f/2.8 1–5× macro lens. A Godox MF12 flash was used as the light source. Images of the morphological details were taken either using a Canon 5D Mark IV digital camera in conjunction with a Mitutoyo Plan NIR 10 lens and a Godox MF12 flash, or with a Nikon SMZ25 stereoscopic microscope and a Nikon DS-Ri2 microscope camera. Zerene Stacker (v. 1.04) was used for image stacking. All images were improved and grouped into plates in Adobe Photoshop CS5 Extended.

Morphological terminology and the format for the descriptions follow Jiang and Wang (2021). The following abbreviations are used in the text: **HW**—width of head across compound eyes; **PL**—length of pronotum along the midline; **PW**—maximum width of pronotum; **EL**—length of elytra along the suture; **EW**—maximum width of elytra; **CL**—the sum of PL + EL.

## ﻿Taxonomy

### ﻿ Zaitzevia fodingshanus

Taxon classificationAnimaliaColeopteraElmidae

Jiang & Chen sp. nov.

15126497-F19F-5E12-B3D0-D4043247CBBF

https://zoobank.org/F38C7F86-8082-472C-B9E9-2FF45C1ADED9

**Figure 1.**
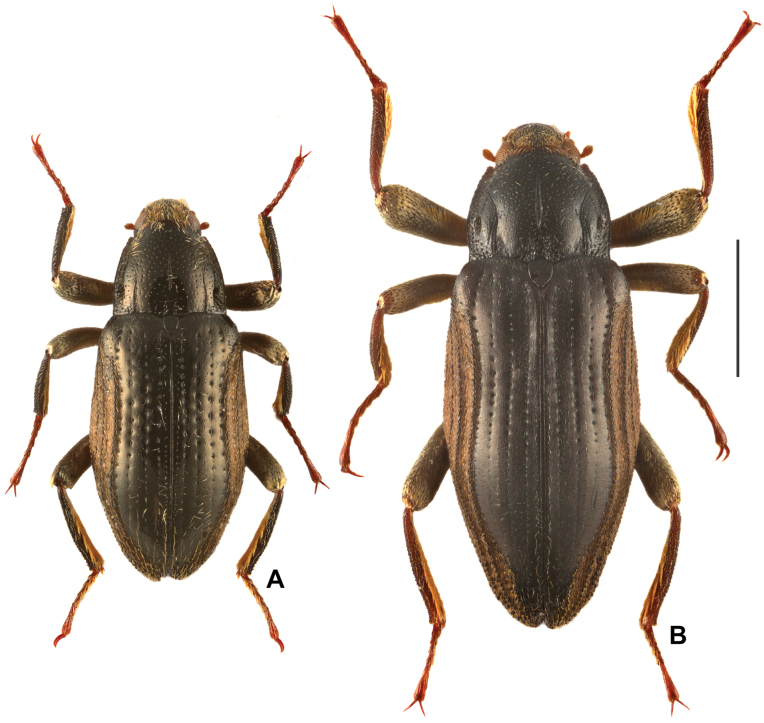
Dorsal habitus of *Zaitzevia* species, males. A. *Zaitzevia fodingshanus* sp. nov., holotype; B. *Zaitzevia lipingae* sp. nov., holotype. Scale bar: 1 mm.

**Figure 2.**
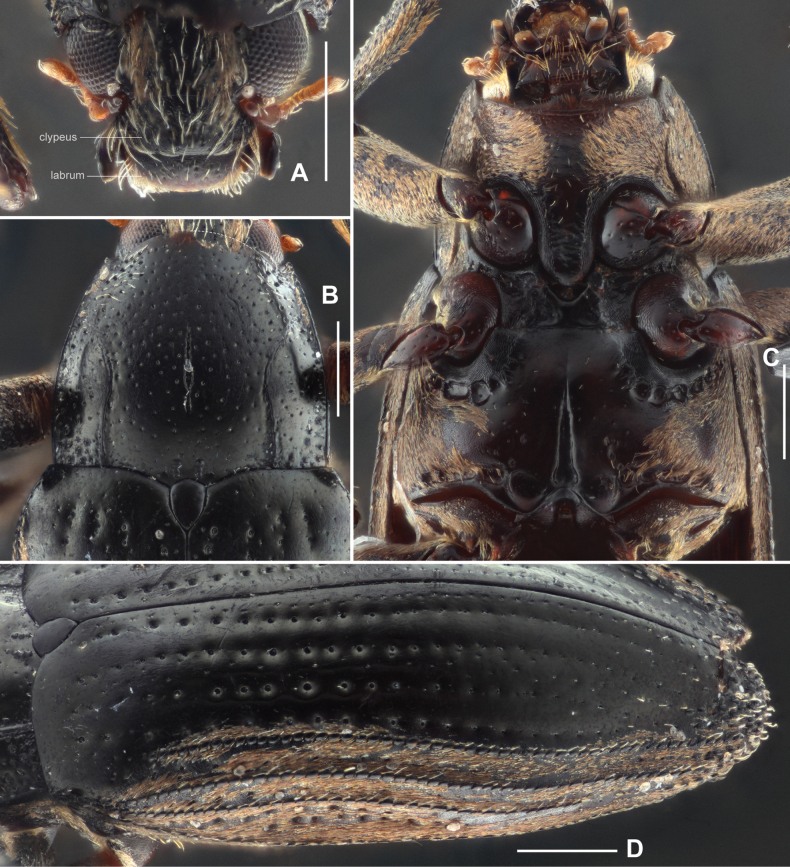
Diagnostic features of *Zaitzevia fodingshanus* sp. nov., holotype. A. Head, dorsal view; B. Pronotum, dorsal view; C. Prosternal process and metaventrite; D. Elytra. Scale bars: 0.5 mm.

**Figure 3.**
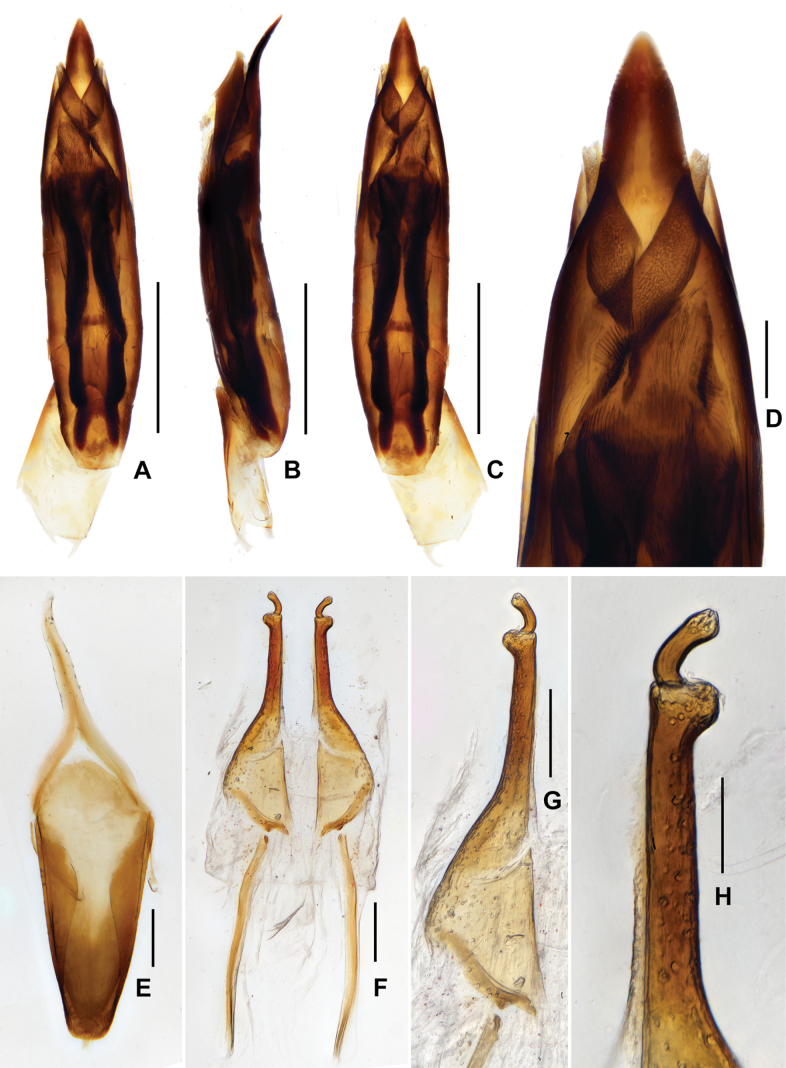
Diagnostic features of *Zaitzevia fodingshanus* sp. nov., holotype. A. Aedeagus, ventral view; B. Ditto, lateral view; C. Ditto, dorsal view; D. Ditto, apex of median lobe, dorsal view; E. Ternite IX; F. Ovipositor; G, H. Apical part of ovipositor. Scale bars: 0.5 mm (A–C); 0.1 mm (D–G); 0.05 mm (H).

Figs 1A, 2, 3 (佛顶山寥溪泥甲)

#### Type material.

(4 ♂♂, 4 ♀♀): **Holotype**: **China**: • ♂, labeled ‘China: Guizhou, Tong ren City (铜仁市), Shiqian County (石阡县), Ganxi Township (甘溪乡), near Fuyan Village (扶堰村), Fodingshan National Nature Reserve (佛顶山国家级自然保护区), an unnamed stream, 27°20'56"N, 108°2'22"E; H: ~850 m, 15.07.2025, Ri-Xin Jiang leg.’ (GUGC). **Paratypes**: • 3 ♂♂, 4 ♀♀, with the same label data as the holotype (GUGC).

#### Description.

**Male.** Body elongately elliptical (Fig. 1A), black, with tarsi, tarsal claws and antennae reddish brown, tibiae brown. Dorsal surface punctate and shiny, covered with sparse short setae. Plastron setae are confined to following areas: head (both dorsal and ventral surface, except discal portion; Fig. 2A), prosternum (Fig. 2C), outer part of elytra (include epipleura; Fig. 2D), outer parts of mesoventrite, metaventrite (Fig. 2C), abdomen (except median part) and surface of femora (Fig. 1A).

***Head*** (Fig. 2A) wider than long, dorsal surface covered with dense short setae and sparse large punctures, each puncture bearing a long seta, short setae absent at discal portion, the interspaces between the punctures about 1–2 times the diameters of punctures. Clypeus evenly punctate with large punctures and covered with long, sparse setae, anterior portion microreticulate. Labrum transverse, shorter and slightly narrower than clypeus, covered with large punctures and long setae at lateral and apical margin, basal portion microreticulate, anterior margin weakly curved and anterolateral angles rounded. Antenna short, slightly clavate, with eight antennomeres, antennomere I slightly longer than wide, with several short setae; antennomere II about as long as antennomere I, distinctly expanded, covered with several long setae, apical margin circled with short setae; antennomere III longer than wide; antennomeres IV–VII strongly transverse; antennomere VIII elliptical, elongate and strongly expanded, apex covered with long, dense setae.

***Pronotum*** (Fig. 2B) slightly wider than long, widest near basal 2/5. Anterior margin arcuate with angles moderately produced and acute. Lateral margins finely curved. Basal margin trisinuate, emarginate before scutellum, posterior angles obtuse. Surface shiny, finely covered with large punctures, each puncture bearing a long seta, several pairs of small granules located at the middle of base of pronotum. Longitudinal impression short and shallow, longer than 1/3 length of pronotum, basal half wider than apical half; sublateral carinae extending from base to the middle of pronotum, apical 1/2 curved, lateral parts of sublateral carinae distinctly convex. Prosternal process (Fig. 2C) with rounded apex, disc without plastron setae, surface distinctly wrinkled.

***Elytra*** (Fig. 2D) about twice as long as wide, nearly subparallel in basal 1/2, surface shiny and covered with long, sparse setae. Each elytron with granulate carinae on strial intervals 5, 7, and 8; other intervals flat. Area from interval 5 to lateral margin covered with short, dense setae. Hind wings well developed.

***Mesothorax*** (Fig. 2C), transverse, surface of disc hairless, with irregular impressions, middle of anterior portion hidden by prosternal process, sides of disc partly covered with plastron setae. Metaventrite (Fig. 2C): surface of disc smooth, anterior portion of disc covered with small, sparse punctures, each bearing a short seta, posterior portion of disc covered with several large and round punctures, sides of disc partly covered with plastron setae. Median sulcus distinct, extending in posterior c. 2/3, narrower and shallower from base to apex, base of median sulcus wide and deep. Areas along coxal cavities and posterior margin of metaventrite with a series of anomalous impressions.

***Disc of ventrites*** I–IV and anteriorly middle of ventrite V shiny, covered with small, sparse punctures, without plastron setae; other areas of ventrites covered with plastron setae. Apical area of ventrite V granulated, apical margin distinctly emarginate at middle.

***Legs*** simple, femora swollen, surface covered with plastron setae; inner side of distal halves of tibiae with cleaning fringes; tarsi slightly shorter than tibiae; tarsal claws simple and strong.

***Aedeagus*** (Fig. 3A–D), elongate, nearly symmetrical, apex of median lobe acutangulus, with two pairs of sclerites; a pair of them located at apical 1/8, wide and curved, becoming narrower from base to apex; another pair of sclerites much longer, about 2/3 length of median lobe. Parameres short, not fused with median lobe. Sternite IX (Fig. 3E) with a tuft of short setae at middle of apical margin, paraproct with base curved.

**Measurements** (*N* = 4): CL: 2.53–2.83 mm; HW: 0.47–0.51 mm; PL: 0.73–0.79 mm, PW: 0.83–0.87 mm; EL: 1.80–2.04 mm, EW: 1.13–1.18 mm.

**Female**: externally similar to the male, apex of sternite VIII rounded. Ovipositor as in Fig. 3F–H, stylus weakly curved at base, apex with several short finger-like sensilla; apex of coxite roundly broadened at outer margin, with several short and curved sensilla; valvifer longer than coxite, fibula weakly curved. Measurements (*N* = 4): CL: 2.56–2.74 mm; HW: 0.48–0.54 mm; PL: 0.71–0.78 mm, PW: 0.83–0.86 mm; EL: 1.81–1.98 mm, EW:1.11–1.25 mm.

#### Distribution.

China: Guizhou Province.

#### Biology.

All adults were collected from a small ravine stream (Fig. 6A–C).

#### Etymology.

The specific epithet refers to the type locality, Fodingshan National Nature Reserve; the name is treated as an adjective.

#### Comparative diagnosis.

*Zaitzevia fodingshanus* sp. nov. is more or less similar to *Z. tangliangi* Jiang & Wang, 2021 from Hubei Province and *Z. yingzuijieensis* Jiang & Chen, 2023 from Hunan Province. All three species share a similar habitus, e.g., a smaller body size, and a shallow and short longitudinal impression of the pronotum. The new species can be easily distinguished from *Z. tangliangi* and *Z. yingzuijieensis* by the base of the pronotum with several pairs of small granules at middle (vs. with a pair of small foveae in the same position in *Z. tangliangi* and *Z. yingzuijieensis*) and the very different form of the aedeagus (aedeagus much slenderer and the anterior pair of sclerites much smaller in *Z. tangliangi* and *Z. yingzuijieensis*; Jiang and Wang 2021; Jiang and Chen 2023).

### ﻿ Zaitzevia lipingae

Taxon classificationAnimaliaColeopteraElmidae

Jiang & Chen sp. nov.

1A0A6F88-EFDB-53D5-8A48-18B8CECE0D29

https://zoobank.org/66420E7D-B98F-44DE-B9EA-8684FE03CB4C

**Figure 4.**
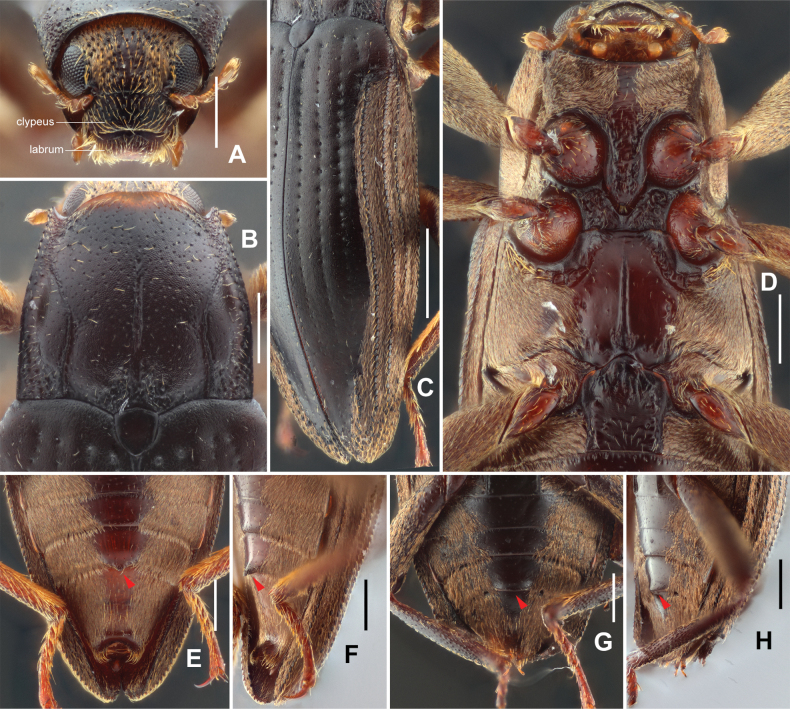
Diagnostic features of *Zaitzevia lipingae* sp. nov., paratype. A. Head, dorsal view; B. Pronotum, dorsal view; C. Elytra; D. Prosternal process and metaventrite; E. Abdomen, male, dorsal view; F. Ditto, lateral view; G. Abdomen, female, dorsal view; H. Ditto, lateral view. Scale bars: 1 mm (C, D); 0.5 mm (A, B, E–H), Note: red arrow of E–H, projection of ventrite IV.

**Figure 5.**
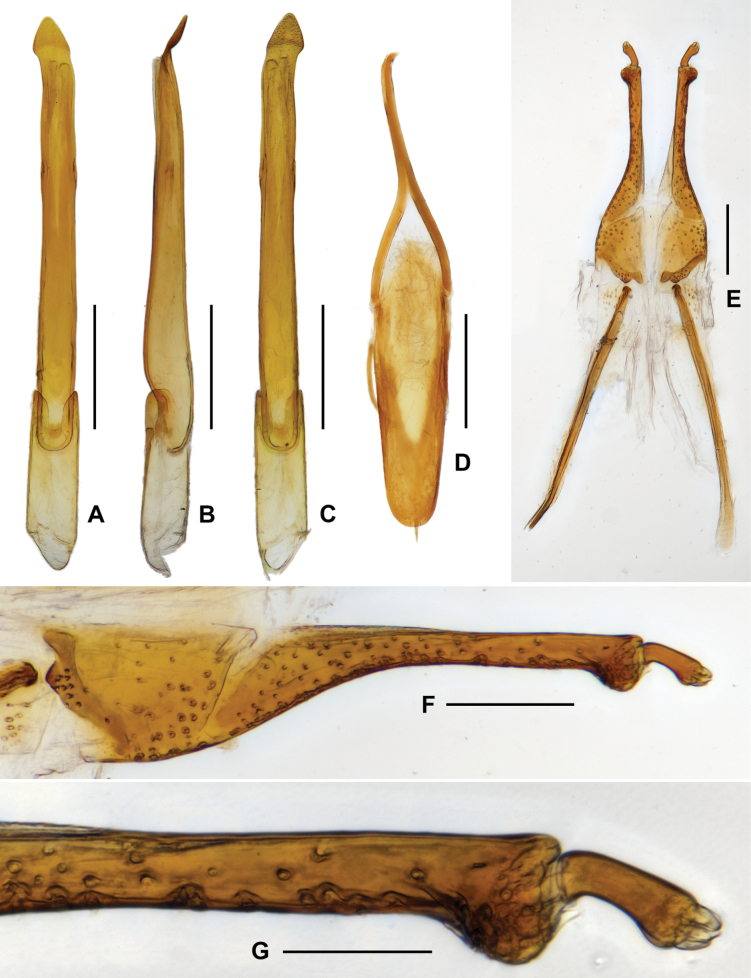
Diagnostic features of *Zaitzevia lipingae* sp. nov., paratype. A. Aedeagus, ventral view; B. Ditto, lateral view; C. Ditto, dorsal view; D. Sternite IX; E. Ovipositor; F, G. Apical part of ovipositor. Scale bars: 0.5 mm (A–D); 0.1 mm (E, F); 0.05 mm (G).

Figs 1B, 4, 5 (李氏寥溪泥甲)

#### Type material.

(3 ♂♂, 2 ♀♀): **Holotype**: **China**: • ♂, labeled ‘China: Yunnan, Bao’an City (保安市), Longyang District (隆阳区), Lujiang Town (潞江镇), near Nankang Village (赧亢村), an unnamed stream, 24°50'46"N, 98°45'38"E; H: ~2000 m, 30.07.2023, Ping Li & Lan Jia leg.’ (GUGC). • **Paratypes**: 2 ♂♂, 2 ♀♀, with the same label data as the holotype (GUGC).

#### Description.

**Male.** Large species, body elongately elliptical (Fig. 2B), black with tibiae, tarsi, tarsal claws and antennae reddish brown, femora dark brown. Dorsal surface punctuated and frosted, covered with sparse setae. Plastron setae are confined to following areas: head (both dorsal and ventral surface, except middle part of frons, labrum and clypeus; Fig. 4A), prosternum (Fig. 4D), outer part of elytra (include epipleura; Fig. 3C), outer parts of mesoventrite, metaventrite (Fig. 4D), abdomen (except median part) and surface of femora (Fig. 4E–H).

***Head*** (Fig. 4A), wider than long, dorsal surface (except middle part of frons, labrum and clypeus) covered with plastron setae and large, sparse punctures, each puncture bearing a long seta, the interspaces between the punctures about 1–1.5 times diameter of punctures. Clypeus without plastron setae, evenly punctate with large punctures and covered with long, sparse setae. Labrum transverse, shorter and slightly narrower than clypeus, covered with big punctures and long bristles at apical half, anterior margin almost straight, anterolateral angles rounded, basal 1/4 without puncture or seta. Antenna short, slightly clavate, with eight antennomeres, antennomere I slightly longer than wide, with several short setae; antennomere II slightly longer than antennomere I, strongly expanded, covered with several long setae, apical margin circled with short setae; antennomere III longer than wide; antennomeres IV–VII strongly transverse; antennomere VIII elliptical, elongate and strongly expanded, apex covered with dense long setae.

***Pronotum*** (Fig. 4B) about as long as wide, widest at base. Anterior margin arcuate with angles moderately produced and acute. Lateral margins finely curved. Basal margin trisinuate, emarginate before scutellum, posterior angles nearly orthogonal. Surface coarse, finely covered with large punctures, each puncture bearing a long seta, punctures at anterior and posterior portions smaller than those at discal parts; surface near apical angles granulated. Longitudinal impression distinct but shallow, about 1/3 length of pronotum, widest at middle; sublateral carinae from base to about 2/3 of the pronotum, apical 3/4 curved. Prosternal process (Fig. 4D) with rounded apex, disc distinctly wrinkled, finely covered with small punctures, each puncture bearing a short seta, lateral sides of prosternal process microreticulated.

***Elytra*** (Fig. 4C) more than twice as long as wide, subparallel in basal 3/5, surface coarse, lateral margins crenulated. Each elytron with granulate carinae on strial intervals 5, 7, and 8; other intervals flat. Area from interval 5 to lateral margin covered with short setae. Hind wings well developed. Apical portion of elytra distinctly granulated.

***Mesothorax*** (Fig. 4D), transverse, surface of disc covered with sparse short setae, and irregular impressions, middle of anterior portion hidden by prosternal process, sides of disc partly covered with plastron setae. Metaventrite (Fig. 4D), disc shiny, covered with small, sparse punctures, each bearing a long seta, without plastron setae, sides covered with plastron setae. Median sulcus long and distinct, extending from posterior margin to c. 4/5 of metasternum, widest at base and becoming narrower anteriorly, base of median sulcus with a deep impression. Areas along coxal cavities and posterior margin with a series of shallow and anomalous impressions.

***Disc of ventrites*** I–IV and anteriorly middle of ventrite V shiny, covered with sparse small punctures, without plastron setae; other areas of ventrites covered with plastron setae. Posterior margin of ventrite IV (Fig. 4E–F) with a small triangular projection at middle. Apical area of ventrite V granulated, apical margin distinctly emarginate at middle.

***Legs*** simple, femora swollen, surface covered with plastron setae; inner half of tibiae with cleaning fringes; tarsi slightly shorter than tibiae; tarsal claws simple.

***Aedeagus*** (Fig. 5A–C), very slender and elongate, median lobe nearly asymmetrical, apical portion curved, with apex nearly triangular. Sternite IX (Fig. 5D) with apical margin rounded, with a tuft of short setae at middle.

**Measurements** (*N* = 3): CL: 3.12–3.44 mm; HW: 0.58–0.62 mm; PL: 0.88–0.97 mm, PW: 0.98–1.05 mm; EL: 2.20–2.56 mm, EW: 1.31–1.46 mm.

**Female**: externally similar to the male, projection of ventrite IV (Fig. 4G–H) weaker than in male, rounded, apex of sternite V rounded. Ovipositor as in Fig. 5E–G, stylus curved at base, apex with several finger-like sensilla; apex of coxite roundly broadened at outer margin, with several short sensilla; valvifer longer than coxite, fibula weakly curved at middle, base weakly expanded. Measurements (*N* = 2): CL: 3.04–3.10 mm; HW: 0.52–0.56 mm; PL: 0.84–0.86 mm, PW: 0.93–0.96 mm; EL: 2.20–2.24 mm, EW: 1.32–1.34 mm.

#### Distribution.

China: Yunnan Province.

#### Biology.

**Figure 6.**
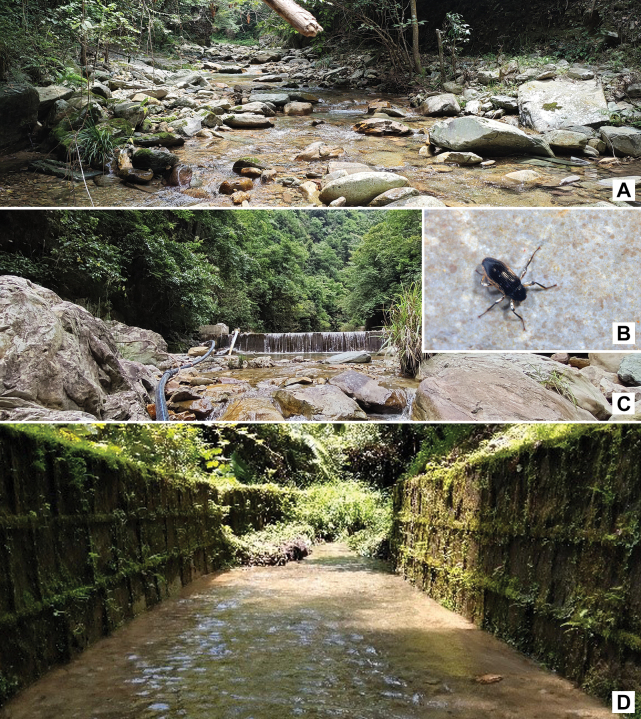
Habitat of *Zaitzevia* species. A, C. General environment of the type locality of *Z. fodingshanus* sp. nov.: Fodingshan National Nature Reserve; B. Living adult of *Z. fodingshanus* sp. nov.; D. General environment of the type locality of *Z. lipingae* sp. nov.

All adults were collected from a small ravine stream (Fig. 6D).

**Figure 7.**
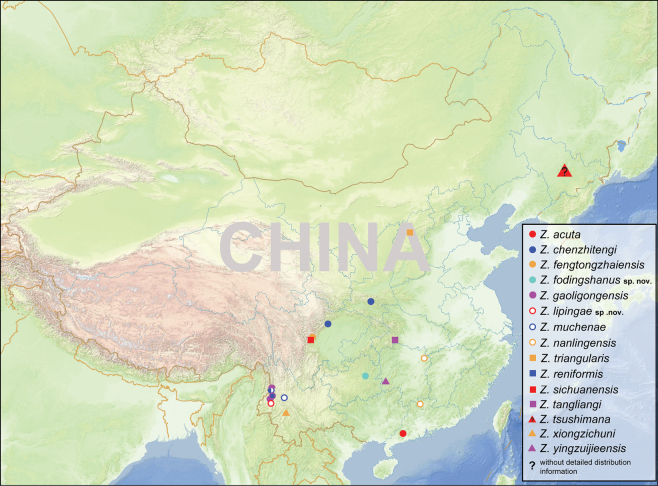
Distributional map of *Zaitzevia* species from the Chinese mainland.

#### Etymology.

The specific epithet “*lipingae*” honors our friend and colleague Dr Ping Li (Guizhou University), one of the collectors of this new species.

#### Comparative diagnosis.

*Zaitzevia lipingae* sp. nov. can be readily distinguished from all other known *Zaitzevia* species by the presence of a distinct projection on ventrite IV and the very long and slender aedeagus.

##### ﻿List of Chinese *Zaitzevia* species

1. *Zaitzevia acuta* Bian & Hu, 2024 (Guangdong)

2. *Zaitzevia babai* Nomura, 1963 (Taiwan)

3. *Zaitzevia chenzhitengi* Jiang & Wang, 2020 (Sichuan, Shaanxi, Yunnan)

4. *Zaitzevia fengtongzhaiensis* Jiang & Chen, 2023 (Sichuan)

5. *Zaitzevia fodingshanus* Jiang & Chen sp. nov. (Guizhou)

6. *Zaitzevia formosana* Nomura, 1963 (Taiwan)

7. *Zaitzevia gaoligongensis* Bian & Zhang, 2022 (Yunnan)

8. *Zaitzevia lipingae* Jiang & Chen sp. nov. (Yunnan)

9. *Zaitzevia muchenae* Bian & Zhang, 2022 (Yunnan)

10. *Zaitzevia nanlingensis* Bian & Hu, 2024 (Guangdong, Hunan)

11. *Zaitzevia parallela* Nomura, 1963 (Taiwan)

12. *Zaitzevia triangularis* Peng, Bian & Wang, 2024 (Shanxi)

13. *Zaitzevia reniformis* Bian & Zhang, 2022 (Yunnan)

14. *Zaitzevia sichuanensis* Jiang & Chen, 2023 (Sichuan)

15. *Zaitzevia tangliangi* Jiang & Wang, 2021 (Hubei)

16. *Zaitzevia tsushimana* Nomura, 1963 (Jilin; Japan; Korea; Russia)

17. *Zaitzevia xiongzichuni* Jiang & Wang, 2020 (Yunnan)

18. *Zaitzevia yingzuijieensis* Jiang & Chen, 2023 (Hunan)

### ﻿Updated key to *Zaitzevia* species from the Chinese mainland (after Jiang and Chen 2023)

**Table:** 

1	Large species, CL > 3 mm	**2**
–	Smaller species, CL < 3 mm	**8**
2	Surface of elytra distinctly wrinkled; aedeagus short and strong, apex of median lobe of aedeagus characteristically arrowhead-like	***Z. chenzhitengi***
–	Surface of elytra shiny or only weakly wrinkled; aedeagus much slender	**3**
3	Posterior margin of ventrite IV with a small triangular projection at middle	***Z. lipingae* sp. nov.**
–	Posterior margin of ventrite IV without projection	**4**
4	Surface of pronotum shiny	***Z. xiongzichuni***
–	Surface of pronotum coarse or weakly wrinkled	**5**
5	Median lobe of aedeagus curved at middle	***Z. sichuanensis***
–	Median lobe of aedeagus not curved at middle	**6**
6	Apex of median lobe of aedeagus widely triangular and strongly curved dorsally	***Z. muchenae***
–	Apex of median lobe of aedeagus not widely triangular, and not or weakly curved dorsally	**7**
7	Body larger, CL ≈ 3.5 mm, apex of median lobe of aedeagus acute	***Z. fengtongzhaiensis***
–	Body smaller, CL ≈ 3.0 mm, apex of median lobe of aedeagus blunt	***Z. triangularis***
8	Longitudinal impression of pronotum extends from base of pronotum	***Z. tsushimana***
–	Longitudinal impression of pronotum not contacted with base of pronotum	**9**
9	Longitudinal impression of pronotum long, longer than 1/2 length of pronotum	**10**
–	Longitudinal impression of pronotum short, less than 1/2 length of pronotum	**11**
10	Median lobe of aedeagus short, less than twice length of phallobase	***Z. gaoligongensis***
–	Median lobe of aedeagus long, longer than twice length of phallobase	***Z. nanlingensis***
11	Body length ≤ 2 mm	**12**
–	Body length > 2 mm	**13**
12	Median lobe of aedeagus strongly curved in lateral view, apex rounded	***Z. reniformis***
–	Median lobe of aedeagus weakly curved in lateral view, apex acute	***Z. acuta***
13	Sublateral carinae of pronotum long, about 1/2 length of pronotum, apical half distinctly curved	***Z. fodingshanus* sp. nov.**
–	Sublateral carinae of pronotum short, about 1/3 length of pronotum, nearly straight	**14**
14	Disc of prosternal process wrinkled, apex of median lobe of aedeagus nearly symmetrical	***Z. tangliangi***
–	Disc of prosternal process shiny, apex of median lobe of aedeagus distinctly asymmetrical	***Z. yingzuijieensis***

## Supplementary Material

XML Treatment for Zaitzevia fodingshanus

XML Treatment for Zaitzevia lipingae

## ﻿Additional information

### Use of AI

No use of AI was reported.

### Author ORCIDs

Zhi-Min Chang https://orcid.org/0000-0002-7247-9706

### Data availability

All of the data that support the findings of this study are available in the main text.
